# Association of Natural Intake of Dietary Plant Sterols with Carotid Intima–Media Thickness and Blood Lipids in Chinese Adults: A Cross-Section Study

**DOI:** 10.1371/journal.pone.0032736

**Published:** 2012-03-07

**Authors:** Ping Wang, Yu-ming Chen, Li-ping He, Chao-gang Chen, Bo Zhang, Wen-qiong Xue, Yi-xiang Su

**Affiliations:** 1 Guangdong Provincial Key Laboratory of Food, Nutrition, and Health, School of Public Health, Sun Yat-sen University, Guangzhou, Guangdong, People's Republic of China; 2 Department of Clinical Nutrition, the Second Affiliated Hospital of Sun Yat-sen University, Guangzhou, Guangdong, People's Republic of China; University of Tor Vergata, Italy

## Abstract

**Background:**

Many studies showed a moderate cholesterol-lowering effect of plant sterols (PS), but increased circulating PS might be atherogenic. We evaluated the associations between natural dietary intake of PS and carotid intima–media thickness (IMT) and serum lipids.

**Methodology/Principal Findings:**

This community-based cross-sectional study included 1160 men and 2780 women aged 31–75 years. Dietary intakes were assessed using a food-frequency questionnaire. The IMTs at the common, bifurcation and internal carotid artery segments, and fasting serum total (TC), LDL (LDLc) and HDL (HDLc) cholesterol, and triglycerides (TG) were determined. After adjusting for potential covariates, multivariate analysis showed a dose-dependent inverse association of total PS intake with serum TC, LDLc, non-HDLc in women (*P*<0.001) and in men (*P*<0.05). As compared to the lowest quartile of PS intake (<206 mg/d), the multivariate-adjusted means of TC, LDLc and non-HDLc in the highest quartile of PS intake (447 mg/d) decreased by 5.0%, 6.2% and 6.5% in women (*P*<0.005), and by 6.4%, 7.1% and 6.7% (*P*>0.05) in men. Although the IMTs tended to be lower with greater intake of dietary PS, only small differences in the left internal IMT between the highest and lowest groups were observed among men (−7.6%) and women (−5.1%) (*P*<0.05). The multivariate analysis showed no significant mean differences among the PS groups in HDLc, TG and IMTs at other studied sites among men and women (all *P*>0.05).

**Conclusions:**

Greater PS consumption from natural diets is associated with lower serum total, LDL, non-HDL cholesterol and with thinner left internal IMT in women and men.

## Introduction

Low-density lipoprotein (LDL) is the major atherogenic lipoprotein. It is the primary target in the reduction of serum cholesterol and in the prevention of cardiovascular diseases (CVD) [1]. A reduction in LDL cholesterol (LDLc) of 10% would reduce the incidence of coronary heart disease (CHD) by 10–20% [1]. Plant sterols (PS) are a group of non-nutritive but bioactive compounds naturally present in all plant origin foods. They are structurally similar to cholesterol and can inhibit cholesterol absorption. Many randomized controlled trials (RCTs) have confirmed that PS added in different foodstuff can significantly reduce total cholesterol and LDLc. A recent meta-analysis including 84 RCTs suggests that 2.15 g/d PS added in fat matrices lowers LDLc concentration by 8.8% [2].

The normal Western-type diet contains 200–400 mg/d PS [3]. The dietary consumption of PS is much lower than those (1–3 g/d) administered in the above-mentioned RCTs [2]. To date, few studies have examined the effects of PS at such a low-dose in natural diets on blood cholesterol. A weak inverse association between natural intake of dietary PS and serum cholesterol concentrations was observed in two cross-sectional studies [4], [5], but not in a controlled feeding study with 459 mg/d PS for 4 weeks [6]. More studies are needed to clarify the effect at low doses in natural diets.

The hypocholesterolemic activity of PS implied their potential efficacy on reducing risk of CVDs. However, epidemiological studies observed conflicting results regarding circulating PS and CVD risks. Many studies found that elevation of plasma or serum PS levels was associated with increased the risk of CVDs [7], [8], and with deposition of those sterols in atheromatous tissues [8]. On the other hand, other studies showed that in physiological range, higher plasma PS levels were associated with lower CVD risk and mortality or not adversely related to CVD risk [9]–[11]. Previous studies suggested that the amount of dietary consumption of PS accounted for very small percentage of variations (4%) in the concentration of circulating PS [12]. The circulating PS cannot be a good marker of the exposure in the body for dietary PS. And the effects of dietary PS cannot be evaluated by determining circulating PS and their association with CVD risk factors. To date, however, most studies examined the cardiovascular effect of circulating but not dietary PS. Little is known about the effects of PS intake from natural diets on atherosclerosis or CVD risks in general population.

Because of the above inconsistent results and limited evidence on the association of natural dietary intake of PS and blood cholesterol and CVD risks, this study aims to examine the hypothesis that greater naturally dietary intake of PS improves plasma cholesterol and atherosclerosis.

## Methods

### Ethics Statement

The Ethics Committee of School of Public Health of Sun Yat-sen University approved the study. Written informed consent was obtained from all individuals at the enrollment.

### Study population

In this community-based cross-sectional study, 4170 apparently healthy Guangzhou residents aged between 31 and 75 year-old were recruited from communities in urban Guangzhou, China between October 2005 and June 2009. Exclusion criteria included subjects who reported having previously confirmed diabetes, CVDs, dyslipidemia, cancers or using medication known to affect lipid metabolism or PS supplements within the three months before the enrollment. 230 subjects were further excluded because of missing data of blood lipids, height or weight. In total, 3940 participants (1160 men and 2780 women) were included in the analysis.

### Questionnaire interview

#### General information

A structured questionnaire was designed to collect socio-demographic information, history and family history of diseases, medication information, smoking and drinking status. Questionnaires were completed under face-to-face interview by trained staffs. Subjects who were smoking at least one cigarette per day for at least six months were classified as current regular smokers [13].

#### Dietary assessment

The quantitative food frequency questionnaire (FFQ) containing 81 items was used to estimate dietary intakes [14]. Most common foods were listed on the FFQ and grouped into following categories: cereals, legumes and legume products, pork, beef, lamb, chicken, fish, shrimp and crab, eggs, dairy, nuts, mushrooms, vegetables, fruits, beverages, alcoholic beverages, supplements and cooking oils. Cooking oil group included peanut oil, rapeseed oil, corn oil, blend oil and soybean oil. The mean intake of food per day, week, month or year was reported at the face-to-face interviews, using the past 12 months prior to the interview as a reference period. Seasonal foods were weighted by the proportion of year that the food was available. Food pictures and full-scale food portion visual aids for the reference portion sizes were provided as visual aids. Dietary intakes were calculated using the China Food Composition 2004 [15]. Dietary intake of PS (β-sitosterol, campesterol, stigmasterol, β-sitostanol and campestanol) was calculated based on the FFQ data and PS database determined by Han et al. [16]. The PS content of animal products and beverages was considered to be nil. No participants in the present population had ever consumed any PS supplements. The short-term reproducibility of dietary PS intakes was assessed using the FFQ separated by about one month in 191 randomly selected from our participants. The correlation coefficients (r) for the short-term reproducibility were 0.436 (*p*<0.001) for total PS, 0.451 (*p*<0.001) for β-sitosterol, 0.401 (*p*<0.001) for campesterol, 0.176 (*p* = 0.019) for stigmasterol, 0.502 (*p*<0.001) for β-sitostanol and 0.497 (*p*<0.001) for campestanol, respectively.

#### Physical activity

24-hour physical activity questionnaire containing 19 items was used to estimate daily physical activity. Physical activities were grouped into activities at work, after work and exercise. Average hours and minutes spent on different activities were reported at the face-to-face interview. Energy expenditure (except sleeping) was calculated according to metabolic equivalent (MET) intensity code [17], [18], activity hours and body weight.

### Measurements of anthropometric indices

Height and weight were measured in light clothing without shoes. Body mass index (BMI) was calculated by weight (kg)/height (m)^2^. Two consecutive measurements of blood pressure were taken from right arm after each subject had been sitting for at least 10 min. The average of the two blood pressures was used for the subsequent analysis.

### Measurement of Carotid intima–media thickness (IMT)

IMT was measured bilaterally at the far wall of the artery by using a high-resolution, 7.0–12.0 MHz linear-array transducer system (Aplio TOSHIBA, Japan) using a predetermined, standardized scanning protocol according to the relevant guideline [19]. The subjects were supine with slight hyperextension and rotation of the neck in the direction opposite the probe. Three segments were measured: the distal common carotid artery segment (CCA) (1 cm proximal to dilation of the carotid bulb), the bifurcation segment (BIF) (1 cm proximal to the flow divider), and the proximal internal carotid artery segment (ICA) (1 cm section of the internal carotid artery immediately distal to the flow divider). The inner and outer walls of carotid artery were scanned longitudinally to assess the best incidence to obtain a clear image. B-mode images at the diastolic phase of the cardiac cycle were recorded by professionals of the Ultrasound Departments of the First and the Second Affiliated Hospitals of Sun Yat-sen University and blinded from subject identity and all the other study parameters. On a longitudinal, two-dimensional ultrasound image of the carotid artery, the far walls of the carotid artery are displayed as two bright white echogenic lines separated by a hypoechogenic space. The wall thickness was manually measured using computer assistance with electronic caliper. Plaques (defined as focal thickening of >1.5 mm) were avoided in the measurement of IMT. The site-specific reliability coefficients based on test-retest using a new image in 91 randomly selected subjects on the same day were estimated as 0.72 and 0.68 for the mean carotid far wall IMT at the common carotid arteries and carotid bifurcation, respectively.

### Blood lipids analysis

12-h fasting venous blood was collected. Serum was separated within two hours and stored at −80°C till tests. Serum total cholesterol (TC), LDL cholesterol (LDLc), HDL cholesterol (HDLc) and triglycerides (TG) were measured by using Hitachi 7600-010 automatic analyzer. The coefficient of variation for lipid measurements was 2.17% (at 5.03 mmol/L TC), 2.86% (at 1.14 mmol/L TG). 3.47% (at 1.70 mmol/L HDLc), 4.67% (at 2.65 mmol/L LDLc).

### Statistical analysis

Data from men and women were analyzed separately. The continuous covariates, such as dietary intakes of, energy, total fat, saturated fat, unsaturated fat to saturated fat ratio, cholesterol and fiber, and physical activity, were converted to standard normal Z-score by gender and age (tertile), and the Z-scores were used in the following analyses. Study participants were categorized into quartiles according to total PS intake from natural diets by gender and age. Mean differences in serum lipids and IMT among the PS quartiles were examined by using univariate and multivariate analyses of variance (ANOVA and ANCOVA). Pair-wise comparisons were done using Bonferroni test. Categorical variables were analyzed with chi-square test. Potential confounding factors including age, BMI, waist circumference, smoking status, menopausal status in women, physical activity, dietary intakes of total energy, total fat, saturated fat, unsaturated fat to saturated fat ratio, cholesterol and fiber were adjusted for in the multivariate model. Analyses were performed with SPSS 16.0 for Windows (SPSS, Inc., Chicago, USA).

## Results

### Characteristics of the participants

Table 1 shows the characteristics of the study subjects. In total, the study included 3940 participants, of which 1160 were male with a mean age of 57.6 y and 2780 were female with a mean age of 55.7 y. Average intake of total dietary PS in the present population was 316±123 mg/d (88.3% of sterol and 11.7% of stanol). Men had higher PS intake (330±129 mg/d) than women (311±120 mg/d) (*p* = 0.007).

**Table 1:** Characteristics of study participants.

	Male			Female		
	Mean	SD	N	Mean	SD	N
Age, year	57.6	6.2	1160	55.7	5.6	2780
BMI, kg/m^2^	23.7	2.9	1155	23.1	3.2	2756
Waist circumference, cm	85.9	8.6	1159	80.8	8.8	2779
Smoker, %	52.2		1160	0.8		2780
Dietary intakes, per day						
Energy, kcal	2115	591	1160	1817	536	2780
Protein, g	87.7	29.6	1160	79.0	29.1	2780
Protein, % total energy	16.6	3.2	1160	17.4	3.5	2780
Fat, g	75.8	33.0	1160	68.8	30.7	2780
Fat, % total energy	31.8	8.1	1160	33.5	7.9	2780
Carbohydrate, g	284	85	1160	233	71	2780
Carbohydrate, % total energy	54.0	9.7	1160	51.9	8.3	2780
Fiber, g	15.0	7.9	1160	14.6	9.0	2780
Cholesterol, mg	427	211	1160	397	198	2780
SFA, g	17.1	9.0	1160	17.2	8.6	2780
(MUFA+PUFA)/SFA	3.02	1.45	1160	3.23	1.57	2780
Total plant sterols, mg	330	129	1160	311	120	2780
Total plant sterols, mg/MJ	38.1	13.1	1160	41.8	14.5	2780
Blood lipids, mmol/L						
TC	5.17	0.92	1160	5.55	1.04	2780
LDLc	3.47	0.83	1160	3.63	0.91	2780
HDLc	1.27	0.31	1160	1.48	0.35	2780
TC-HDLc	3.91	0.88	1160	4.07	0.99	2780
Total triglycerides	1.82	1.35	1160	1.57	1.04	2780
Carotid IMT, mm						
Left CCA	0.75	0.24	1011	0.68	0.18	2509
Left BIF	1.05	0.33	1011	0.92	0.25	2509
Left ICA	0.69	0.19	755	0.62	0.15	2006
Right CCA	0.73	0.19	856	0.67	0.15	2214
Right BIF	1.00	0.30	856	0.90	0.24	2214
Right ICA	0.69	0.20	755	0.62	0.16	2005

**Abbreviations:** BMI: body mass index; CCA: common carotid artery segment; BIF: bifurcation segment; HDLc: high density lipoprotein cholesterol; ICA: internal carotid artery segment; IMT: Carotid intima-media thickness; LDLc: low density lipoprotein cholesterol; MUFA: monounsaturated fatty acids; PUFA: polyunsaturated fatty acids; SFA: saturated fatty acids; TC: total cholesterol; TG: total triglycerides.

Greater intake of PS was associated with dietary intakes of energy and almost all nutrients, such as protein, carbohydrate, total fat, saturated fat, fiber, etc. After adjustment for energy, higher total PS intake was associated with greater intake of carbohydrate, fiber and with lower intake of total fat and saturated fatty acids in both men and women. In men, greater total PS intake was associated with lower smoking rate. (Data not shown)

### Food sources of dietary PS intake

β-sitosterol was the predominant PS in Chinese diets. Mean intakes of β-sitosterol, campesterol, stigmasterol, β-sitostanol and campestanol were 202±126 (63.1%), 48±21 (14.9%), 34±12 (10.7%), 31±13 (9.7%), 6±3 (2.0%) mg/d, respectively. 34.7%, 27.4%, 27.4%,7.3% and 3.2% of total PS were from cereals, vegetables and fruits, cooking oil, nuts, legumes and legume products, respectively. (**Table 2**)

**Table 2:** Food sources of dietary plant sterol intake in men and women (mg/d).

	Cereals	Legumes	Vegetables and fruits	Nuts	Cooking oil	Total intake
Total plant sterol	111±48	10±13	88±49	23±38	88±89	317±127
β-sitosterol	62.4±27.3	6.1±7.7	57.9±32.5	17. 8±28.9	58.1±57.2	202.3±82.3
Campesterol	19.7±8.7	2.2±2.7	8.6±5.4	2.0±3.1	15.2±17.4	47.7±20.9
Stigmasterol	7.7±3.9	1.5±2.0	17.0±9.5	1.3±2.2	6.8±5.4	34.3±12.4
β-sitostanol	16.5±7.2	0.4±0.7	4.6±2.8	2.0±3.2	7.6±9.1	31.1±12.8
Campestanol	5.0±2.8	0.1±0.2	0.7±0.4	0.2±0.3	0.3±1.0	6.3±3.2

Values are mean ± SD.

### Dietary PS and serum lipids

Overall, univariate analyses of variance showed that total PS intake was dose-dependently and inversely associated with serum TC, LDLc and non-HDLc in both men and women. After adjusting for potential covariates such as, age, BMI, waist circumference, smoking status, menopausal status in women, physical activity, dietary intakes of total energy, total fat, saturated fat, unsaturated fat to saturated fat ratio, cholesterol and fiber, the differences in mean values of TC, LDLc and non-HDLc became more pronounced in women and not in men. Dose-dependent inverse associations between dietary PS intake and these lipids (TC, LDLc and non-HDLc) was remained in both women (p-trend<0.001) and men (p-trend<0.05). As compared to the lowest quartile of PS (206 mg/d), the multivariate-adjusted means of TC, LDLc and non-HDLc in the highest quartile of PS intake (447 mg/d) decreased by 5.0% (p<0.001), 6.2% (p = 0.002) and 6.5% (p<0.001) in women, and by 6.4%, 7.1% and 6.7% (all p>0.05) in men. The multivariate analysis showed no significant mean differences among the PS groups in HDLc and TG among men and women (all p>0.05). (**Table 3** **&** **Table 4**)

**Table 3:** Mean (SD) of blood lipids (mmol/l) and IMT (mm) by quartiles of dietary consumption of PS.

	Quartile 1			Quartile 2			Quartile 3			Quartile 4			ANOVA	
	Mean	SD	N	Mean	SD	N	Mean	SD	N	Mean	SD	N	*P*_1_	*P*_2_
Male														
TC	5.29	0.92	288	5.18	0.88	290	5.16	0.95	293	5.07	0.90†	289	0.031	0.004
LDLc	3.59	0.84	288	3.47	0.83	290	3.43	0.82	293	3.38	0.83†	289	0.017	0.002
HDLc	1.28	0.30	288	1.29	0.34	290	1.26	0.32	293	1.24	0.28	289	0.185	0.041
TC–HDLc	4.01	0.87	288	3.89	0.86	290	3.90	0.89	293	3.83	0.90	289	0.090	0.022
TG	1.77	1.12	288	1.87	1.40	290	1.85	1.49	293	1.78	1.38	289	0.743	0.974
IMT														
Left CCA	0.75	0.27	253	0.76	0.24	251	0.73	0.22	253	0.74	0.22	254	0.741	0.489
Left BIF	1.03	0.34	253	1.06	0.31	251	1.07	0.33	253	1.05	0.34	254	0.545	0.387
Left ICA	0.70	0.20	188	0.66	0.18	187	0.72	0.21‡	189	0.66	0.16	191	0.003	0.407
Right CCA	0.75	0.20	204	0.73	0.18	215	0.73	0.19	223	0.73	0.20	214	0.704	0.260
Right BIF	0.98	0.27	204	0.98	0.27	215	1.05	0.36	223	1.00	0.26	214	0.083	0.176
Right ICA	0.68	0.17	188	0.67	0.16	187	0.71	0.22	189	0.69	0.23	191	0.326	0.339
Female														
TC	5.68	1.09	692	5.54	1.06	696	5.49	1.04††	696	5.49	0.96††	696	0.001	<0.001
LDLc	3.74	0.96	692	3.65	0.93	696	3.59	0.90†	696	3.55	0.85†††	696	<0.001	<0.001
HDLc	1.49	0.35	692	1.47	0.35	696	1.47	0.33	696	1.48	0.35	696	0.583	0.486
TC–HDLc	4.19	1.03	692	4.07	1.00	696	4.01	0.99†	696	4.02	0.93††	696	0.003	<0.001
TG	1.54	0.92	692	1.60	1.07	696	1.57	1.13	696	1.58	1.03	696	0.771	0.613
IMT														
Left CCA	0.68	0.20	631	0.67	0.17	626	0.67	0.17	616	0.67	0.16	636	0.890	0.459
Left BIF	0.92	0.26	631	0.92	0.26	626	0.90	0.23	616	0.93	0.24	636	0.260	0.878
Left ICA	0.62	0.17	502	0.62	0.15	503	0.62	0.16	499	0.60	0.14	502	0.133	0.103
Right CCA	0.67	0.16	530	0.68	0.16	561	0.66	0.14	560	0.66	0.16	563	0.362	0.178
Right BIF	0.90	0.23	530	0.90	0.24	561	0.89	0.25	560	0.90	0.24	563	0.898	0.958
Right ICA	0.62	0.18	502	0.62	0.16	502	0.62	0.15	499	0.62	0.15	502	0.998	0.890

P1: P for group difference; P2: P for trend.

†,††,††† : Compared with Quartile 1, P<0.05, P<0.01, P<0.001;

‡ : P<0.05, compared to Quartile 3.(Bonferroni). Abbreviations: refer to **Table 1**.

**Table 4:** Covariate-adjusted mean (SEM) of blood lipids and IMT by quartiles of dietary consumption of PS.

	Plant sterol intake				%Differ-ence*	ANCOVA	
	Quartile 1	Quartile 2	Quartile 3	Quartile 4		*P*_1_	*P*_2_
Men							
*Serum lipids, mmol/l*							
LDL-C	3.67±0.07	3.49±0.06	3.38±0.06†	3.41±0.07	−7.08	0.036	0.022
TC	5.31±0.08	5.12±0.07	5.05±0.07	4.97±0.08	−6.40	0.068	0.012
HDL-C	1.257±0.024	1.25±0.019	1.20±0.019	1.19±0.024	−5.33	0.132	0.036
TC-HDLC	4.054±0.078	3.865±0.063	3.849±0.063	3.783±0.077	−6.68	0.138	0.043
TG	1.733±0.098	1.630±0.079	1.751±0.079	1.598±0.097	−7.79	0.790	0.579
*IMT, mm*							
Left CCA	0.783±0.020	0.800±0.016	0.769±0.016	0.780±0.020	−0.38	0.627	0.692
Left BIF	0.995±0.028	1.037±0.023	1.043±0.023	1.005±0.028	1.01	0.372	0.810
Left ICA	0.706±0.017	0.669±0.014	0.722±0.014	0.652±0.017†	−7.65	0.002	0.230
Right CCA	0.763±0.017	0.752±0.013	0.738±0.014	0.730±0.016	−4.33	0.612	0.187
Right BIF	0.976±0.024	0.964±0.019	1.005±0.019	0.976±0.023	0.00	0.482	0.751
Right ICA	0.686±0.017	0.673±0.014	0.699±0.014	0.683±0.017	−0.44	0.609	0.842
Women							
*Serum lipids, mmol/l*							
LDL-C	3.756±0.040	3.650±0.035	3.594±0.035†	3.522±0.039††	−6.23	0.002	<0.0001
TC	5.728±0.046	5.545±0.040†	5.470±0.040†††	5.440±0.045†††	−5.03	<0.0001	<0.0001
HDL-C	1.491±0.014	1.470±0.012	1.471±0.012	1.477±0.014	−0.94	0.653	0.550
TC-HDLC	4.237±0.043	4.075±0.037†	3.999±0.037†††	3.963±0.042†††	−6.47	<0.0001	<0.0001
TG	1.540±0.044	1.600±0.039	1.576±0.039	1.594±0.044	3.51	0.764	0.539
*IMT, mm*							
Left CCA	0.681±0.008	0.674±0.007	0.675±0.007	0.671±0.008	−1.47	0.864	0.464
Left BIF	0.929±0.011	0.921±0.010	0.902±0.010	0.918±0.011	−1.18	0.312	0.346
Left ICA	0.629±0.008	0.617±0.007	0.622±0.007	0.597±0.008†	−5.09	0.045	0.027
Right CCA	0.668±0.007	0.678±0.006	0.665±0.006	0.661±0.007	−1.05	0.299	0.370
Right BIF	0.907±0.012	0.900±0.010	0.893±0.010	0.895±0.011	−1.32	0.874	0.488
Right ICA	0.619±0.008	0.622±0.007	0.623±0.007	0.620±0.008	0.16	0.976	0.961

* : Difference between Quartile 4 and Quartile 1.

ANCOVA: analysis of covariance. Covariates adjusted for: age, BMI, waist circumference, physical activity, smoking status, alcohol drinking, menopause status (in women), dietary intakes of energy, total fat, saturated fatty acids, ratio of unsaturated fat to saturated fat, cholesterol, fiber. *P*_1_: *P* for group difference; *P*_2_: *P* for trend.

†,††,††† : Compared with Quartile 1, *P*<0.05, *P*<0.01, *P*<0.001 (Bonferroni).

Abbreviations: refer to **Table 1**.

### Dietary PS and carotid intima–media thickness (IMT)

Although the IMTs tended to decrease with greater dietary intake of PS, only small differences were observed at the left internal IMT in men and women (p<0.05). The mean values of the left internal IMT decreased by 7.6% and 5.1% in the highest quartile as compared to the lowest quartile of PS (p<0.05). No significant difference was observed in the common IMT and bifurcation IMT among the four PS groups in both men and women. (**Table 3** **&** **Table 4**)

## Discussion

In this large community-based cross-sectional study, a moderate dietary intake of total PS was found (317 mg/d) in this population. We generally found a dose-dependent inverse association between PS intake from natural diet and serum TC, LDLc and non-HDLc in middle-aged and elderly Chinese. Our findings suggest that greater intake of PS in natural diet may be favorable in the prevention of CVDs in Chinese.

### Plant sterols and blood cholesterol

It has been established that high dose PS can significantly reduce blood cholesterol in RCTs [2]. For LDLc management, National Cholesterol Education Program Adult Treatment Panel III (NCEP ATP-III) induced the maximal dietary therapy in which PS/stanols is the most powerful component for LDLc reduction [1], [20]. However, it was still uncertain whether PS was effective at low doses from natural diets due to limited evidence. In our study, an increase of 264 mg/d PS in men and 241 mg/d PS in women from the lowest to the highest group was associated with 5.3%, 6.7% and 6.9% (women, p<0.01), and 3.6%, 4.2% and 3.3% (men, p>0.05) of decreases in serum TC, LDLc and non-HDLc after adjustment for potential covariates, respectively. Similar positive results were observed in two large cross-sectional studies. In the European Prospective Investigation into Cancer and Nutrition (EPIC-Norfolk) study, a 289 mg/d (in men), 281 mg/d (in women) increase of total PS intake correlated to 4.1%, 2.4% decrease in serum TC and 3.5%, 3.0% decrease in LDLc, respectively [4]. Another study including 37 150 men and 40 502 women observed 2.6% (in men), 3.5% (in women) lower TC and 3.1% (in men), 3.2% (in women) lower LDLc in highest quintile compared to the lowest quintile of PS [5]. The different results might be due to specificity of the population and different covariates adjusted for in the multivariate models. In a recent observational study conducted in Spain with a small sample size and relatively high dietary PS intake (513 mg/d on average), serum LDLc decreased significantly across increasing tertiles (545 vs. 428 mg/d) of PS intake from natural diet [21]. The efficacy in the observational studies seemed to be stronger than that from RCTs. A meta-analysis including 84 clinical trials showed that approximate 700 mg/d PS was required to achieve 4.5% reduction in LDLc [2]. Greater intake of PS in the control group, higher baseline blood concentration, short intervention (12 to 182 d), co-existing dietary factors (e.g., carbohydrate, vegetable and unsaturated fatty acids) and ceiling effect might partially explain the small efficacy in RCTs as compared to observational studies [2]. In our study, the inverse associations between dietary PS intake and serum TC, LDLc and non-HDLc was greatly attenuated in men after adjusting for potential covariates. It should be noted that cigarette smoking which is considered to be one of six major modifiable risk factors for CVDs was inversely related to dietary PS intake in men in this population. Serum TC, LDLc and TG are increased by smoking, while levels of HDLc are decreased [22]. The effects of smoking on serum lipids might partially explained why the statistical significance of inverse associations between PS intake and serum TC, LDLc and non-HDLc was attenuated in men, after adjusting for potential covariates.

The mechanism of hypocholesterolemic activity of PS was extensively summarized in a recent review [23]. It has long been considered that PS displaces cholesterol from mixed micelles due to their greater affinity for micelles. Consequently, they decrease intestinal cholesterol absorption and a higher fecal excretion of cholesterol and its metabolites. However, this mechanism could not explain why single or multi-dose supplementation of PS resulted in similar decrease in blood LDLc [24]. Recent studies suggested that PS might lower LDLc by upregulating intestinal cholesterol efflux transporters and receptor-mediated lipoprotein cholesterol uptake in response to the reduced supply of exogenous cholesterol [23].

In the present study, we did not observe significant difference in HDLc among the PS groups. Previous studies showed inconsistent associations between dietary PS intake and serum HDLc concentration in human studies. Blood HDLc reduction was observed in men in EPIC Norfolk study (−6.3%) [4] and in Sweden women (−2.2%) [5] (quintile: V vs. I). However, a large number of RCTs had examined the PS on blood HDLc. Supplemental plant sterols or stanols in general produced little or no change in HDLc [2]. A meta-analysis even noted a trend towards improvement in HDLc in patients with type 2 diabetes [25]. These results suggested that HDLc was unlikely to be decreased by plant sterols themselves. Many dietary factors co-existing with plant sterols might explain partially the differences in HDLc levels.

### Plant sterols and cardiovascular risks

Comparing to the consistent results of cholesterol-lowering, PS have controversial and inconclusive effects on the risk of CVDs. Up to now, it has been much debated whether higher levels of circulating PS might be atherogenic in nonsitosterolemic individuals due to the presumed atherogenic role of elevated plasma phytosterols in sitosterolemic patients [12]. A few studies observed a positive association between increased circulating PS levels and elevated risk of CVDs [7], [8] and deposition of those sterols in atheromatous tissues [8]. However, other studies found elevated circulating PS have null effect or even significantly beneficial effects on CVD risks, and lowered long-term mortality [9]–[11]. The reasons for the between-study heterogeneity were still uncertain. However, the above studies showed that detrimental effects of circulating PS tended to be more frequently found in patients with hypercholesterolemia or CVDs, in populations with greater but not moderate elevation of circulating phytosterols or with phytosterols supplements but not from natural diets [7], [8], [26]. Mutations in ABCG5 or ABCG8 in sitosterolemic patients characterized by extremely high circulating sitosterol might explain the proatherogenic effects of sitosterolemia [27]. In a nested case-control study among participants of the Spanish EPIC cohort with similar PS intake level (315 mg/d on average) to our study, moderately elevated plasma sitosterol was associated with reduced risk of coronary heart diseases [28].

Many factors, such as analytical methods, gender, Apo E phenotype, ABCG8/G5 phenotype, diabetes mellitus, metabolic syndrome and dietary intake can affect the circulating PS concentrations [12]. Although some studies showed plasma PS levels was significantly increased by supplementation of PS [8], it was estimated that the dietary intake only contributed to 4.03% of variations in circulating PS concentration [12]. To our knowledge, no study reported the association of PS intake from natural diets and cardiovascular risks or atherosclerosis in human.

Carotid intima-media thickness (IMT) is a strong predictor of future vascular events. A recent meta-analysis showed that the relative risks of myocardial infarction and stroke were 1.26 (95% CI, 1.21 to 1.30) and 1.32 (95% CI, 1.27 to 1.38) per 1–SD increase of the common carotid artery IMT [29]. Although many clinical trials had demonstrated the cholesterol-lowering benefits of PS, the association between PS and the progress of atherosclerosis remained unclear. Similar to the effects on blood total and LDL cholesterol, the carotid IMTs tended to be lower with an increase of PS intake, and significant difference was observed in the internal IMT in our subjects. Previous studies showed associations between IMT and CVD risk factors differed in artery sides and segments. ICA IMT was a stronger independent correlate of incident myocardial infarction and prevalent CHD, whereas CCA IMT was a stronger correlate of stroke. BIF IMT was associated with ischemic heart disease risk factors [30]–[32]. Therefore, relationships between IMT and PS intake were evaluated in different sides and segments. Generally consistent with our finding, a recent cross-sectional epidemiological study reported CCA IMT was weakly inversely correlated with serum campesterol/sitosterol-to-cholesterol ration but not with serum campesterol or sitosterol concentration in 583 hospital employees aged 25–60 years without prevalent cardiovascular disease or lipid-modifying medication in Germany [33].

To our knowledge, this is the first large study that examined the association between natural dietary PS consumption and IMT after adjustment for potential confounders, such as age, BMI, menopausal status in women and dietary intakes of energy, cholesterol, saturated fatty acids and fiber. Our findings showed that moderately increased intake of PS from natural diets might be beneficial to prevent the development of atherosclerosis. Besides the cholesterol-lowering effect, previous studies showed that PS could reduce C-reactive protein (CRP) concentration, improve inflammatory markers and endothelial functions [34]. These effects might contribute to the prevention of atherosclerosis.

### Plant sterols intake in food sources

The average intake of PS in the present population was similar to that reported from Mediterranean area with the diet rich in plant-based foods, in which the average PS intake was reported to be between 315 and 375 mg/d [35]–[38]. Cereals and oils represent the two most important sources in both Mediterranean diet and the present diet, but cereals, vegetables and fruits contributed more to dietary total PS intake in the present population as compared with Mediterranean area [37].

### Limitations

The major limitation of this study is the cross-sectional study design which might have limitations in the identification of causal relationship. In the present study, we exclude the subjects who had previously confirmed diabetes, CVDs, dyslipidemia, cancers which might change their dietary habits to avoid any potential inverse causal relationship. Moreover, we included only those subjects who had lived in the Guangzhou (the field site) for five or more years to improve the subject's stability of lifestyle. Previous study showed that adults generally maintain a relatively stable dietary habit over a long period in Scottish middle-aged women [39]. Our previous studies also showed good long-term reproducibility, with 84% and 83% having within one unit quartile agreements in fruit and vegetable intakes [40], and good validity in the dietary assessment by FFQ [14]. These data suggested that the intake of PS assessed by using FFQ might reflect a long-term and stable habitual intake of PS from habitual diets in this population. Another limitation is that we did not evaluate circulating PS due to limited budget, and thus could not examine the association between circulating PS and atherosclerosis in this study population.

### Conclusion

In conclusion, in the present cross-sectional study conducted in Guangzhou, China, higher PS intake from natural diets is inversely related to serum concentrations of total cholesterol, LDL cholesterol and non-HDL cholesterol, and the left internal carotid IMT in both women and men. Our findings add to the existing evidence that greater levels of dietary consumption of PS, at least in the natural dietary intake range, may be favorable for the prevention of cardiovascular diseases.
